# To resuscitate or not to resuscitate; a question for old age psychiatrists

**DOI:** 10.1192/bjo.2021.113

**Published:** 2021-06-18

**Authors:** Liam Embliss, Mohan Bhat

**Affiliations:** Goodmayes Hospital, North East London NHS Foundation Trust

## Abstract

**Aims:**

The inpatient population of an older adult psychiatric ward will include people with physical and mental health conditions which affect life span and quality of life. Patients may be frail, acutely unwell, or have terminal illnesses such as dementia. It is therefore essential that clinicians review resuscitation status as part of their routine practice. However, we are aware that advanced decision-making – to resuscitate or not to resuscitate – is not routine practice across older adult psychiatric wards in the UK. Our 2017 audit reflected this, demonstrating a very low rate of resuscitation decisions at NELFT.

This re-audit aimed to measure the frequency and quality of resuscitation decisions on an older adult psychiatric ward. We expected improvements in these areas, subsequent to changes implemented from the initial audit. We also sought to identify which patient factors influenced clinicians’ decision-making on resuscitation.

Please note, this audit was completed prior to the COVID-19 pandemic.

**Method:**

In June 2017, an audit of 25 patients admitted to two older adult psychiatric acute wards was completed. In December 2019, a retrospective analysis of the last 25 admissions to one older adult ward was undertaken. Electronic patient notes and DNACPR (Do Not Attempt Cardiopulmonary Resuscitation) orders were examined. The audit measured frequency of resuscitation decisions and quality of documentation against current standards. DNACPR orders were analysed and clinicians were interviewed to identify the reasons for such decisions.

**Result:**

There was an increase in the number of patients for which resuscitation decisions were made, from 4% in 2017 to 40% (n = 10) in 2019. The majority of patients with a DNACPR decision (n = 8) had a diagnosis of dementia. Prospective quality of life, with this diagnosis, was the most frequent determinant of DNACPR decisions (n = 7). Qualitative analysis indicated that clinicians were more likely to consider a resuscitation decision for patients with an organic disorder rather than functional disorder.

Adequate completion of DNACPR orders was seen in each case. Either the patient, a family member or carer was involved in every decision. The standard for recording decisions on the electronic patient record was not met.

**Conclusion:**

It is good practice to consider resuscitation decisions for patients admitted to older adult psychiatric wards. This re-audit found an improvement in frequency of resuscitation decisions and also revealed differences in decision-making for patients with organic and functional disorders. Implementation of further change is indicated; decision-making can be improved through reflection, teaching, changes to practice, and technologies.

